# Rumen-protected glucose stimulates the secretion of reproductive hormones and the mTOR/AKT signaling pathway in the ovaries of early postpartum

**DOI:** 10.1038/s41598-023-30170-5

**Published:** 2023-02-20

**Authors:** Yan Wang, Chenzhong Jin, Yuzhen Yi, Yihong Hu, Xuefeng Han, Zhiliang Tan, Zheng Wang, Jinhe Kang

**Affiliations:** 1 College of Agriculture and Biotechnology, Hunan University of Humanities, Science and Technology, Hunan, China; 2 CAS Key Laboratory for Agro-Ecological Processes in Subtropical Region, National Engineering Laboratory for Pollution Control and Waste Utilization in Livestock and Poultry Production, Hunan Provincial Key Laboratory of Animal Nutrition and Physiology and Metabolism, South-Central Experimental Station of Animal Nutrition and Feed Science in Ministry of Agriculture, Institute of Subtropical Agriculture, The Chinese Academy of Sciences, Hunan, China; 3 College of Bioscience and Biotechnology, Hunan Agricultural University, Hunan, China

**Keywords:** Hormones, Biochemistry, Zoology, Animal physiology, Gene expression analysis

## Abstract

This study was conducted to determine the response of the reproductive hormones and the mTOR/AKT/PI3K pathway in the ovaries of postpartum dairy cows with dietary rumen-protected glucose (RPG). Twelve Holstein cows were randomly assigned to two groups (n = 6/group): the control group (CT) and the RPG group. Blood samples were collected on d 1, 7, and 14 after calving for the gonadal hormone assay. The expression of the gonadal hormones receptors and PI3K/mTOR/AKT pathways were detected using RT-PCR and Western blot. The RPG addition increased the plasma LH, E2, and P4 concentrations on d 14 after calving and upregulated the mRNA and protein expressions of the ERα, ERβ, 17β-HSD, FSHR, LHR, and CYP17A1 but downregulated StAR expression. Immunohistochemical analysis identified higher expressions of the FSHR and LHR in the ovaries of RPG-fed cows compared to CT cows. Furthermore, the protein expressions of *p*-AKT/AKT and *p*-mTOR/mTOR were significantly increased in the ovaries of RPG-fed cows compared to the CT group, but the addition of RPG did not alter the protein expression of *p*-PI3K/PI3K. In conclusion, the current results indicated that dietary RPG supplementation regulated gonadotropin secretion and stimulated expression of hormone receptors and the mTOR/AKT pathway in the ovaries of early postpartum dairy cows. RPG may be beneficial for the recovery of ovarian activity in post-calving dairy cows.

## Introduction

Ovarian restoration of cows after calving is critical to ensure the next reproductive cycle^1^. However, the early postpartum cows are in negative energy balance (NEB) due to the dual stress of reduced feed intake and increased milk production^2^, and NEB was associated with delayed ovulation^3^. Glucose is a critical nutrient in postpartum dairy cows. It can induce milk synthesis and cell growth in mammary epithelial cells^4^. In addition, the uptake of glucose by ovarian tissue during the estrous cycle in ruminants modulates the ovary function^5^. However, ruminants absorb very little glucose from the diet, as dietary carbohydrates provide glucose precursors in the form of propionate absorbed from the rumen. But glucose can be absorbed directly from the gastrointestinal tract if it bypasses rumen digestion^6^. It can be sufficient to meet the nutritional requirement of milk production^7^. Because direct glucose absorption from the small intestine can be more energy efficient^8^. For example, an infusion of 1,000 g of glucose through the jugular vein can rapidly alter the hormonal and metabolic profiles and improve reproduction in postpartum cows^9^. A more direct way of enhancing the net glucose absorption for ruminants is to feed the rumen-protected glucose (RPG) coated with fat. Our previous studies also confirmed that dietary RPG supplementation replenishes energy, improves milk production, and stimulates the insulin-like growth factor system in the endometrium of early postpartum dairy cows^10,11^.

In ovarian function, the phosphatidylinositol 3-kinase (PI3K)/protein kinase B (AKT)/mechanistic target of rapamycin complex1 (mTOR) pathway plays a key role, including steroidogenesis, granulosa proliferation, corpus luteum survival, and oocyte maturation^12^. The growth and proliferation of granulosa cells are essential for developing ovarian follicles. Steroid hormones are synthesized by steroidogenic enzymes such as cytochrome p450 aromatase (CYP19A1), cytochrome p450 (CYP17A1), and cytochrome P450scc (CYP11A1)^13^. The nutritional status of dairy cows affects fertility by providing nutrients required for gamete development, insulin-like growth factor 1 (IGF-1) production, and luteinizing hormone (LH) in the blood^14^. Indeed, LH stimulates the expression of CYP17A1 in bovine theca cells by activating PI3K/AKT pathway^15^. Follicle-stimulating hormone (FSH) can also activate the PI3K pathway. A delicate interplay has been shown to exist between cAMP/protein kinase A and PI3K signaling in the regulation of steroidogenesis by FSH in rat granulosa cells. At the same time, AKT is known to cause mTOR activation through various mechanisms^16^, synergistically stimulating follicle growth^17^. Our previous studies have also shown that the mTOR/AKT pathway regulates endometrial cell proliferation in early postpartum cows and maybe benefit uterine recovery^11^.

The objective of this study was to determine the effects of supplementing RPG from d -7 ± 2 to 14 postpartum on the secretion of the gonadal hormones (FSH, LH, E2, P4) along with the expressions of their receptors and the mTOR/AKT pathway in the ovaries of the early post-partum dairy cows.

## Results

### Ovary weight index

**Figure 1:**
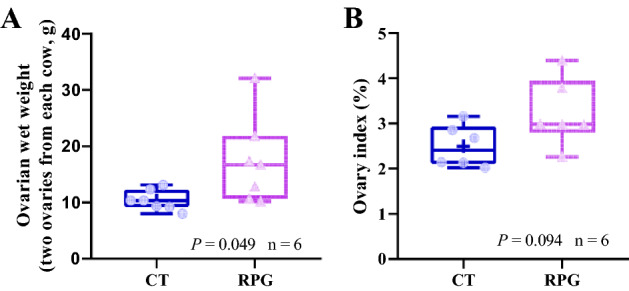
Box and whisker plots of the ovarian weight (**A**) and ovary index (**B**) from postpartum dairy cows that show significant (*P* < 0.05) or a tendency (0.05 < *P* < 0.01) between the control diet and the rumen-protected glucose diet.

The ovarian weight on 14 d postpartum was significantly higher (*P* = 0.049), and the ovary index tended to be higher (*P* = 0.094) among cows in the RPG group as compared to the control diet (CT) group (Fig. 1) but not significantly.

### Postpartum plasma hormones concentrations

**Figure 2:**
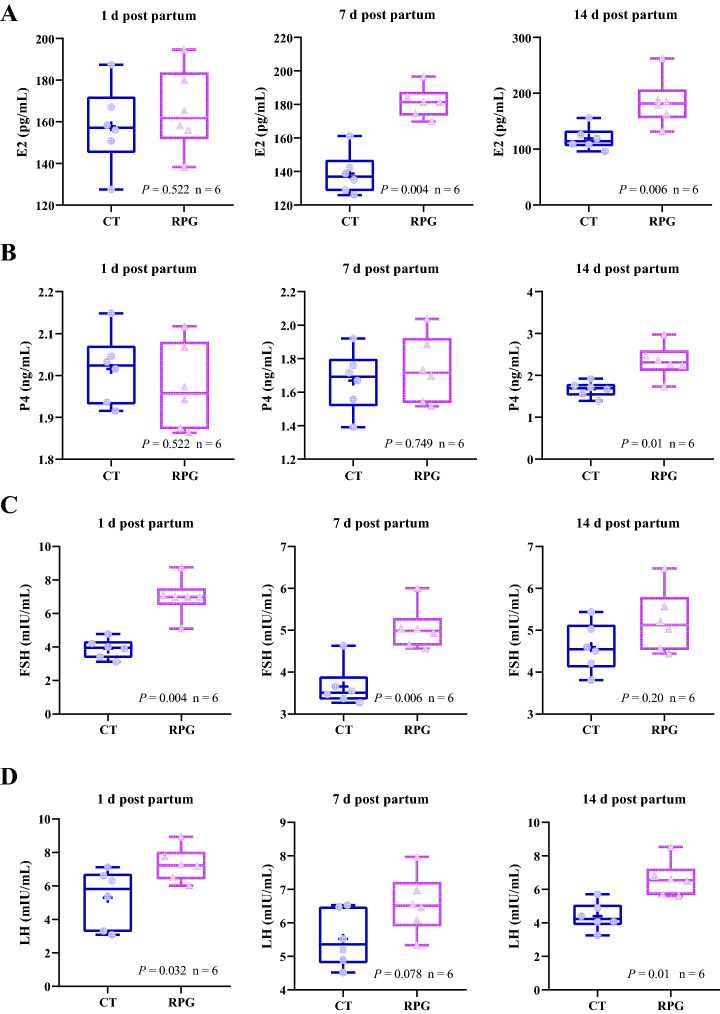
Box and whisker plots of the plasma concentrations of (**A**) E2, (**B**) P4, (**C**) FSH, and (**D**) LH from postpartum dairy cows that show significant (*P* < 0.05) or a tendency (0.05 < *P* < 0.1) between control diet and rumen-protected glucose diet. E2: *P*_Time_ = 0.562, *P*_Group_ = 0.000, and *P*_Time×Group_ = 0.17; P4: *P*_Time_ = 0.002, *P*_Group_ = 0.003, and *P*_Time × Group_ = 0.001; FSH: *P*_Time_ = 0.003, *P*_Group_ = 0.000, and *P*_Time × Group_ = 0.001; LH: *P*_Time_ = 0.228, *P*_Group_ = 0.000, and *P*_Time × Group_ = 0.394.

E2 and LH concentrations were significantly affected by the RPG addition. The E2 concentrations were significantly increased in the RPG group compared with the CT group at d 7 (*P* = 0.004) and 14 (*P* = 0.006) postpartum. They were comparable between the two groups at d 1 postpartum (Fig. 2A). The concentrations of P4 and FSH were significantly affected by the RPG addition (*P*_Group_ = 0.003, *P*_Group_ = 0.000, respectively), the collection time (*P*_Time_ = 0.002, *P*_Time_ = 0.003, respectively), and their interactions (*P*_Time×Group_ = 0.001, *P*_Time×Group_ = 0.001, respectively). Furthermore, the P4 concentrations were comparable between the two groups on d 1 and 7 postpartum. They were significantly increased (*P* = 0.01) in the RPG group compared with the CT group on d 14 postpartum (Fig. 2B). Meanwhile, the FSH concentrations were significantly increased in the RPG group on d 1 (*P* = 0.004) and 7 (*P* = 0.006) postpartum compared with the CT group, and were comparable between two groups at d 14 postpartum (Fig. 2C). The LH concentrations in the RPG group were higher than that in the CT group (Fig. 2D) on d 1 (*P* = 0.032) and d 14 (*P* = 0.01) postpartum. They tended to be increased (*P* = 0.078) in the RPG group at d 7 postpartum compared with the CT group.

### Gene expression profile

**Figure 3:**
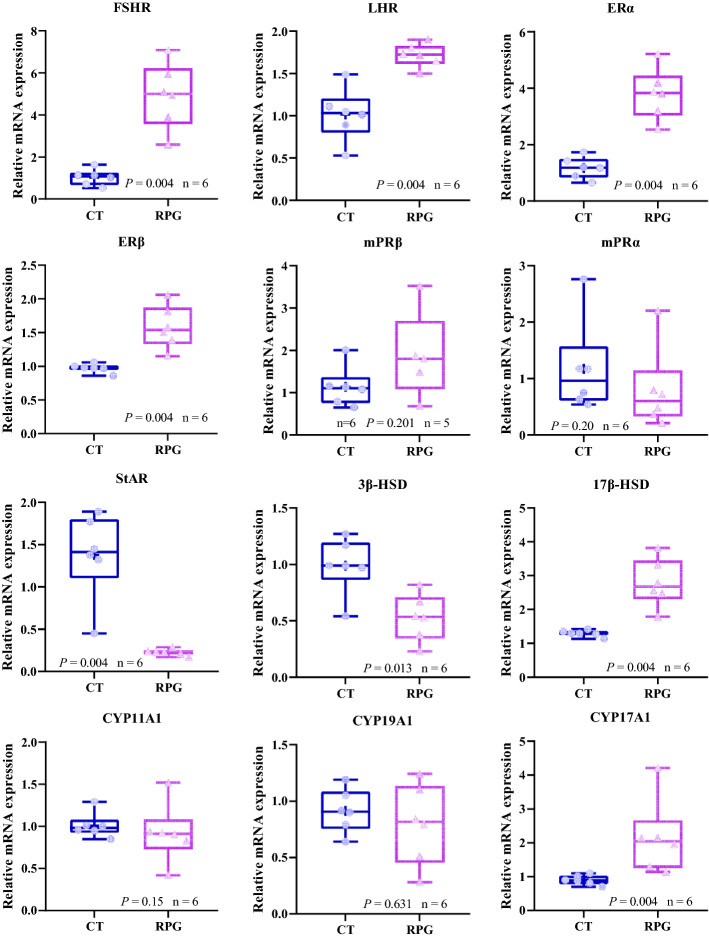
Box and whisker plots of the FSHR, LHR, ERα, ERβ, mPRα, mPRβ, StAR, 3β-HSD, 17β-HSD, CYP11A1, CYP19A1 and CYP17A1 gene expression in ovarian tissue from postpartum dairy cows that show significant (*P* < 0.05) between control diet and rumen-protected glucose diet.

As illustrated in Fig. 3, the FSHR, LHR, estrogen receptor alpha (ERα), estrogen receptor beta (ERβ), 17β-hydroxysteroid dehydrogenase (17β-HSD), and CYP17A1 mRNA expression levels were significantly increased (*P* = 0.004, *P* = 0.004, *P* = 0.004, *P* = 0.004, *P* = 0.004, and* P* = 0.004, respectively) in the ovaries of the RPG group cows compared with the CT group cows. However, there were no differences in the mRNA expression levels of mPRα (*P* = 0.20), mPRβ (*P* = 0.201), CYP11A1 (*P* = 0.15), and CYP19A1 (*P* = 0.631). The mRNA expression levels of 3β-hydroxysteroid dehydrogenase (3β-HSD) and steroidogenic acute regulatory protein (StAR) were significantly decreased (*P* = 0.013 and* P* = 0.004, respectively) in the ovaries of the RPG group cows compared with the CT group cows.

### Protein expression pattern

**Figure 4:**
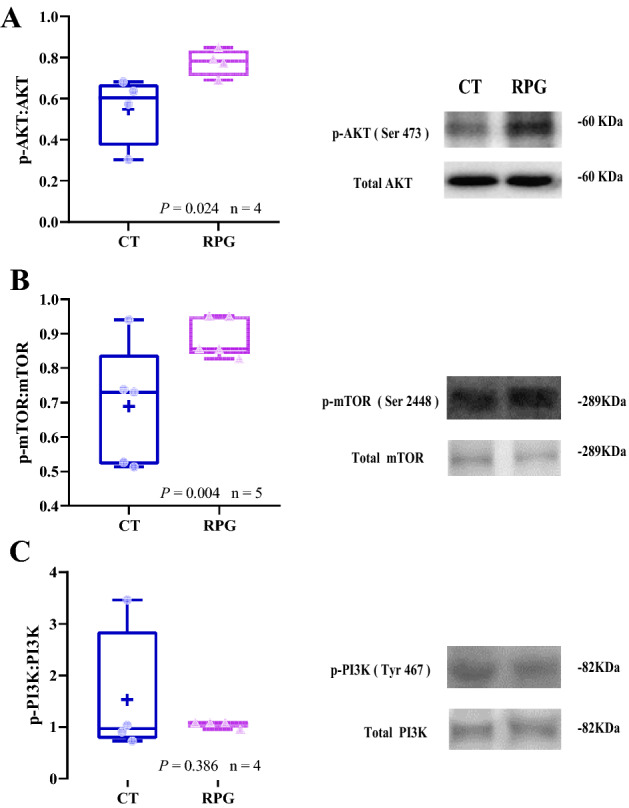
Box and whisker plots of the protein expression ratio of (**A**) p-AKT (Ser473): AKT, (**B**) *p*-mTOR (Ser2448): mTOR, and (**C**) *p-*PI3K (Tyr 467): PI3K proteins in ovarian tissue from postpartum dairy cows that show significant (*P* < 0.01) between control diet and rumen-protected glucose diet. Figure 4 of the original blots/gels is presented in Supplementary Fig. 2.

**Figure 5:**
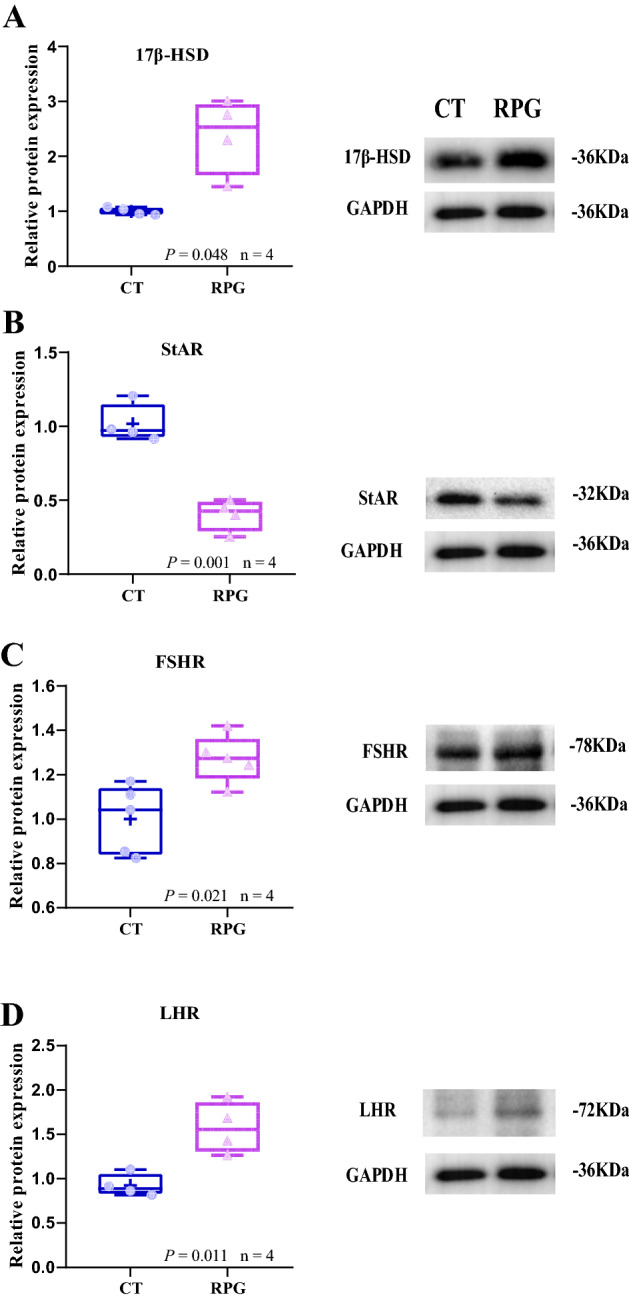

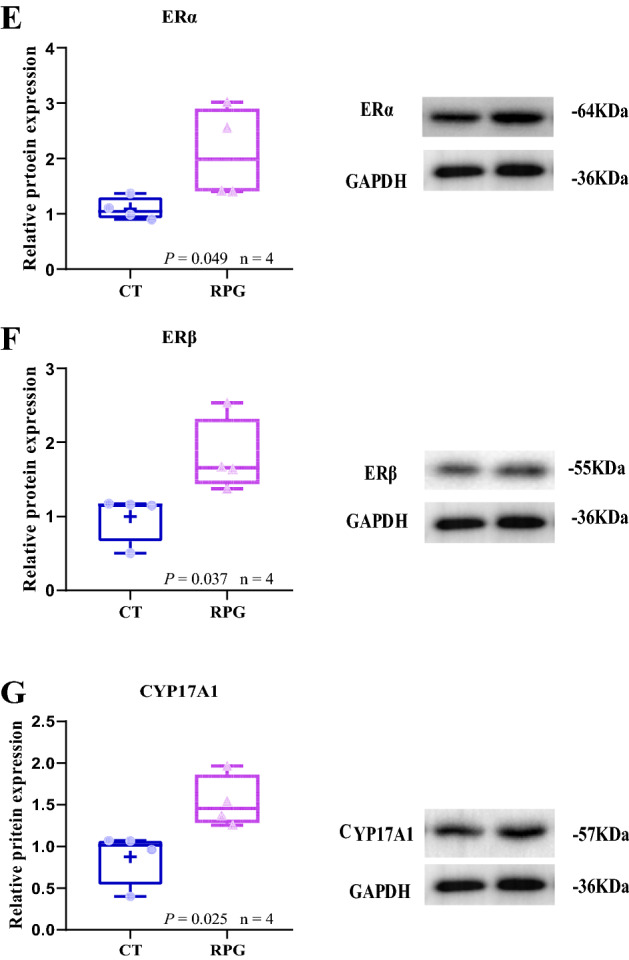
Box and whisker plots of the protein expression of (**A**) 17 β-HSD, (**B**) StAR, (**C**) FSHR, (**D**) LHR, (**E**) ERα, (**F**) ERβ, and (**G**) CYP17A1 in ovarian tissue from postpartum dairy cows show significant (*P* < 0.05) between control diet and rumen-protected glucose diet. Figure 5 of the original blots/gels is presented in Supplementary Fig. 3.

As shown in Fig. 4, the ratios of the protein expression levels of *p*-AKT to total AKT and *p*-mTOR to total mTOR were significantly increased (*P* = 0.024,* P* = 0.004, respectively) in the ovaries of RPG-fed cows compared with the CT group cows. However, the ratio of *p*-PI3K to total PI3K was comparable (*P* = 0.386) between the two groups. Meanwhile, the protein expression levels of 17βHSD, FSHR, LHR, ERα, ERβ, and CYP17A1 were significantly increased (*P* = 0.048, *P* = 0.021, *P* = 0.011, *P* = 0.049, *P* = 0.037, and *P* = 0.025, respectively) in the ovaries of RPG-fed cows versus CT cows (Fig. 5A, C–G). However, the protein expression level of StAR was decreased (*P* = 0.001, Fig. 5B) in the ovaries of RPG-fed cows versus CT cows.

### Ovarian histology

**Figure 6:**
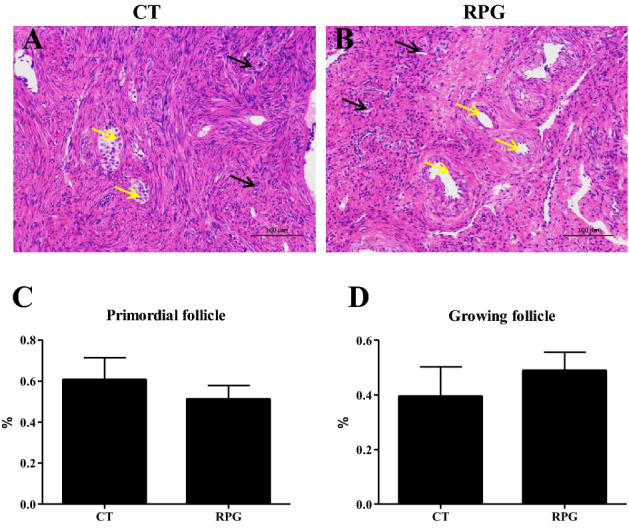
Effects of RPG and CT supplementation on ovarian follicle development. (**A**) Ovary of CT dairy cows. (**B**) The ovary with increasingly growing follicles after RPG treatment. (**C**) The percentage of the primordial follicle in two groups. (**D**) The percentage of growing follicles in two groups. Black arrows represent primordial follicles. Yellow arrows represent growing follicles. Scale bars = 100 μm. Data are presented as the means ± SEM.

In the CT group, visible primordial follicles (Black arrows), growing follicles (Yellow arrows), cuboidal granulosa cells, no follicular atresia, and corpus luteum were observed (Fig. 6A). However, the volume and number of growing follicles were increased in the RPG group compared to the CT group, and there was no newly formed follicular atresia and corpus luteum in the RPG group (Fig. 6B). There was no difference in the percentage of primordial follicles and follicles growing between two groups (Fig. 6C, D; *P* = 0.468, *P* = 0.09, respectively) in the RPG group but not significantly.

### Expression of FSHR and LHR in ovarian tissue determined by immunohistochemistry

**Figure 7:**
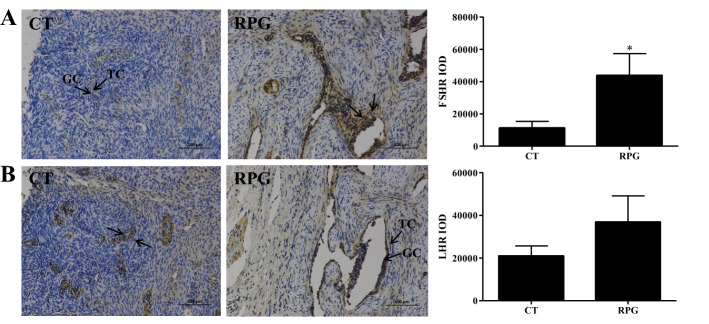
Effects of RPG and CT supplementation on FSHR and LHR expression level in ovarian tissue from postpartum dairy cows. (**A**) Representative micrographs of positive FSHR staining in ovarian tissues. (**B**) Representative micrographs of positive LHR staining in ovarian tissues. The integral optical density (IOD) of FSHR and LHR was calculated by Image-Pro Plus 6.0. B. *IC* interstitial cell, *TC* theca cell, *GC* granulosa cell. Data are presented as the means ± SEM. Compared to the control diet: *P < 0.05.

The FSHR protein expression in ovarian tissues of dairy cows was significantly increased (*P* = 0.046) in RPG cows compared with the CT cows (Fig. 7A). In addition, the LHR protein expression tended to increase (*P* = 0.098) in the RPG-fed cows compared with the CT cows (Fig. 7B) but not significantly.

## Discussion

It is necessary to restore postpartum ovarian activity as soon as possible to achieve normal fertility and shorten the calving interval^18^. The resumption of ovarian activity in postpartum cows depends upon a fully functional hypothalamic-pituitary-ovarian (HPO) axis interaction^19^. The current theory is that metabolites and metabolic hormone act as signals directly on follicles to regulate folliculogenesis through specific ovarian receptors^20^. Previous studies have shown that the LH depletion/repletion cycle in the anterior pituitary is a major limiting factor in early postpartum recovery at 2–4 weeks after calving^21^. LH is essential for follicle development and oocyte maturation^22^. In addition, it is associated with increased hypothalamic sensitivity to the positive feedback effect of estradiol^21^. Usually, estrogens act via two types of receptors (ERα and ERβ), which are selectively stimulated or inhibited, depending upon a balance between ERα and ERβ activities in target organs^23^. Also, the growth rate of the antral follicles is suppressed during pregnancy and even after calving. This inhibition lasted for about 20 days after calving and reduced the frequency of ovulation on the same side of the uterine horn^24^.

It is well known that levels of reproductive hormones and metabolic factors are regulated by nutritional status. The cows' follicular development and oocyte quality were affected by nutrition^11,25^. Among them, glucose is a master regulator of hormones and metabolites that control the reproductive process. Blood glucose concentration in early postpartum cows does not meet the body's needs due to the high energy demand for postpartum recovery and milk synthesis during early lactation. In the present study, the higher plasma concentrations of E2, P4, LH, and the mRNA and protein levels of gonadotrophin response gene (FSHR, LHR) and estradiol synthesis-related genes (ERα, ERβ) in the ovaries of the cows in the RPG group compared to CT group on the 14 d postpartum indicated that RPG addition stimulates the secretion of reproductive hormones, the expressions of hormone receptors in the ovaries of early postpartum dairy cows. A similar result has been reported by Lucy et al.^9^ that the hormonal and metabolic profile of the postpartum cows was rapidly changed when 500 g/d glucose was injected into the cows via the intravenous route. The potential mechanism in which RPG supplementation influences the secretion of reproductive hormones and the expression of their receptors may be that RPG can increase P4 concentration and influence gonadotropin-releasing hormone (GnRH) release from hypothalamic neurons, and both FSH and LH are released from the anterior pituitary in response to GnRH^26^. Furthermore, the ovaries take up glucose during the estrous cycle, which depends on insulin^26^. Our previous studies have shown that plasma insulin levels can be enhanced in postpartum dairy cows fed RPG supplementation^10^. Moreover, feeding dietary starch that promotes glucose levels favors an early return to postpartum ovulation^27^. These results suggested that a glucogenic diet could stimulate the ovary to utilize glucose, improving somatotropic axis synergies with the gonadotropins (FSH, LH, and P4, etc.) in post-partum dairy cows^28^.

Moreover, ovarian steroid hormones regulate follicular growth and atresia. In the ovary, use the steroidogenic genes (CYP19A1, CYP17A1, and CYP11A1) to convert cholesterol to estrogens^20^. In the current study, the mRNA and proteins expressions of 17β-HSD and CYP17A1 (the key gene of P4 and androstenedione secretion)^29^ were upregulated in RPG-fed cows compared with CT cows, although the mRNA and proteins expressions of 3β-HSD and StAR were downregulated and the mRNA expressions of CYP11A1 and CYP19A1 were not affected. These results suggested that the synthesis of gonadotropins may be accelerated and that the expressions of strategic factors were stimulated in cows in the RPG group compared to those in the CT group.

Early recovery of postpartum ovarian activity, which refers to oocyte growth and follicle development, as well as early ovulation, which depends on the regulation of pathways involved in cell proliferation, cell survival, and cell cycle regulation, such as the PI3K/AKT/mTOR signaling pathway^30,31^. LH stimulates AKT phosphorylation, and PI3K/AKT activation is involved in the expression of CYP17A1 mRNA in cultured bovine theca cells. Also, LH is involved in mTOR activation in bovine granulosa cells^32^. It has also been demonstrated that FSH can activate the PI3K/AKT pathway in granulosa cells and cultured follicles, which plays a key role in folliculogenesis, including the activation of follicles and maturation^33^. In the present study, the *p*-AKT/AKT and *p*-mTOR/mTOR expression levels were up-regulated in the ovaries of cows fed an RPG supplementation diet compared to cows fed a CT diet. These results indicated that the AKT/mTOR pathway was activated in the ovaries of RPG-fed cows. Although the percentage of primordial follicles was not changed, an increasing trend in the percentage of growing follicles was observed in RPG-fed cows. This result was consistent with a previous report that rumen bypass starch decreased the maximum number of small follicles and improved ovarian function in high-yielding dairy cows during early lactation^34^. This response may depend on the insulin supply being sufficient for the recruitment of small follicles and the movement of the follicles to the larger^34^. Furthermore, the ovary takes up glucose depending on insulin during the estrous cycle^25^. Our previous study showed that the plasma insulin level was enhanced in postpartum dairy cows supplemented with RPG^10^. Dietary supplementation with RPG may help promote oocyte growth and follicle development in postpartum dairy cows.

Only 12 cows were selected in this study due to the condition limitations of the experimental farm where this study was conducted. The number of animals is a limitation of the results of this study, especially for dairy industries that manage large herds of cows under different management. Further studies with larger numbers of animals are needed to explore the effects of RPG on a larger scale.

But we also calculated the statistical power by using the *GPower* software. Based on the current scenario with 12 cows, the power of E2, P4, FSH, and LH are 0.992, 0.802, 0.999, and 0.964 (Supplementary Fig. 1), respectively, indicating the statistical power of 12 cows was credible.

## Conclusions

**Figure 8:**
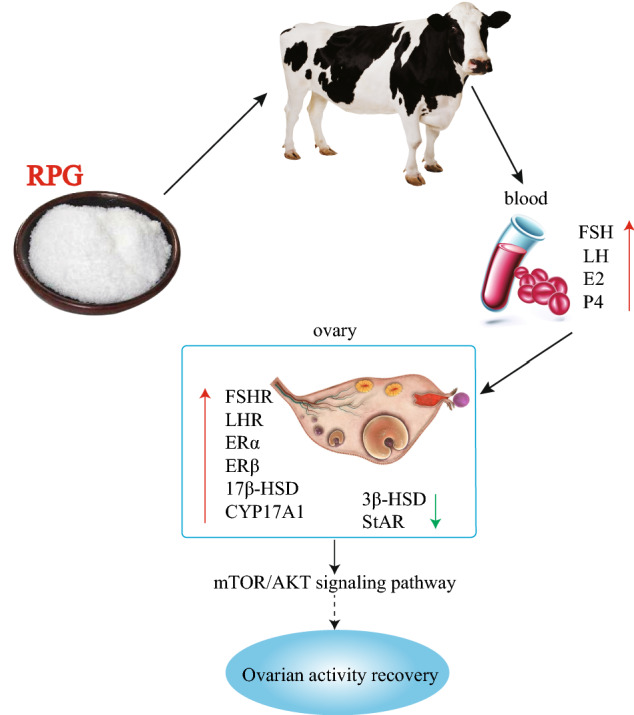
The schematic diagram for RPG improving the ovarian activity recovery in post-natal dairy cows. RPG addition might promote FSH, LH, E2, and P4 binding to their respective receptors, thereby activating the mTOR/AKT signaling pathway and accelerating ovarian activity recovery in post-calving dairy cows.

In summary, our results revealed that dietary RPG supplementation regulated the secretion of gonadal hormones and stimulated the expressions of hormone receptors and the mTOR/AKT pathway in the ovaries of early postpartum dairy cows (Fig. 8).

## Materials and methods

### Ethics approval

The experimental procedures and all methods were carried out in accordance with relevant institutional guidelines and regulations and were approved by the Animal Welfare Committee (Permit No. ISA000257), Institute of Subtropical Agriculture, the Chinese Academy of Sciences, Changsha, China. The study was carried out in compliance with the ARRIVE guidelines (https://arriveguidelines.org) for reporting animal experiments.

### Animals and experimental procedures

Twelve healthy Holstein cows of age (4–5 years), body weight (515 ± 42 kg), milk yield (16.1 ± 3.7 kg/d), parity (2.8 ± 0.4), and expected calving date (June 12 ± 2.6 d) were randomly assigned to 2 treatments (n = 6/group): (1) control diet (CT) group; (2) control diet plus rumen-protected glucose (RPG, 200 g/d per cow) group. The RPG (Yahe Nutrition Technology Co., Ltd., Beijing, China) is manufactured by a patented technique, containing 45% glucose coated with fat (45%). Considering the possible effects of coating fat in RPG, similarly quantified coating fat was supplemented in the CT group with 90 g per cow daily. Twelve cows were housed in 16-row free-stall barns with deep recycled manure bedding in Hunan province, a typical subtropical region in southern China. In addition, pregnant animals in the prepartum pen were frequently monitored by on-farm personnel for imminent signs of parturition. At that time, they were moved into contiguous maternity pens for calving. All cows were fed the same close-up diet from d-21 to the expected parturition and lactation diet from parturition through d-20. The RPG and coating fat were top-dressed on the Total Mixed Rations (TMR) and fed to all treatments from d -7 ± 2 to d 14 twice daily in equal quantities at 7:15 and 14:15 h during summer (June to July). The basal diet was fed ad libitum twice daily at 7:30 and 14:30 h. The ingredients and chemical composition of the diet are shown in Supplementary Table 1. All cows were given free access to water throughout the trial period. The environment temperature was 34 ± 0.35 °C, and the daily average humidity (g/kg) was 940 ± 12.5.

### Sample collection

Blood samples were collected from each cow before the morning feeding using heparinized tubes (18 U/mL) on d 1, 7, and 14 postpartum and immediately stored on ice. Plasma was separated via centrifugation (3000× g, 15 min) at 4 °C and stored at − 80 °C. The cows, approximately on d 22 post-partum, were slaughtered using electrical stunning followed by exsanguination according to the usual practices of the China beef industry^35^. The ovaries (bilateral) were collected from cows within 20 min after slaughter, cleaned up fat and connective tissue, rinsed in RNase-free phosphate buffer, and the wet weight of the ovaries was measured. The ovary index was calculated using the following formula: ovarian index = wet ovarian weight (g)/body weight (kg) × 100%. The unilateral ovary of each cow was divided into quarters, and two quarters were immediately frozen in liquid nitrogen and stored at − 80 °C for RNA isolation and protein extraction. The other two-quarters of the ovary was fixed in 10% buffered formalin for histology and immunohistochemistry analyses. Also, the operator who took the ovary samples was completely blinded to the purpose of the cow treatment.

### Blood hormonal analysis

The concentrations of FSH, LH, E2, and P4 in the plasma of cows were determined by Bovine specific Enzyme-Linked Immunosorbent Assay (ELISA) kits (Cusabio, Wuhan, China) according to the manufacturer’s protocols. The product codes for the FSH, LH, E2, and P4 ELISA kits are CSB-E15856B, CSB-E12826B, CSB-E08173b, and CSB-E08172b, respectively. These kits were validated on plasma from cows. FSH, LH, E2, and P4 assay kits have a detection range of 2–800 mIU/mL, 1.25–100 mIU/mL, 40–1000 pg/mL, and 0.15–70 ng/mL, respectively. FSH, LH, E2, and P4 assay kits have sensitivities of 2 mIU/mL, 1.56 mIU/mL, 40 pg/mL, and 0.2 ng/mL, respectively. Each plasma sample was analyzed in duplicate. Plasma samples from all cows at all time points were measured in one run. The intra- and inter-assay coefficients of variation for E2, P4, FSH, and LH were 5.5 and 6.1, 6.1 and 7. 2, 5.5 and 6.6, 5.1 and 6.1%, respectively.

### Gene expression analysis

Total RNA was isolated using Trizol reagent (Invitrogen, Carlsbad, CA) as described previously^11,36^. Total RNA concentration was quantified with an ND-1000 UV–vis spectrophotometer (NanoDrop Ltd., Wilmington, DE). The integrity of RNA was inspected using 1% agarose gel electrophoresis according to Wang et al.^11^. To eliminate traces of genome DNA, 1 µg total RNA was treated with 5 × gDNA Eraser Buffer, 2.0 μl Eraser Buffer, 1.0 μl gDNA Eraser, and RNase-free DNase I (TaKaRa, Dalian, China) for 2 min at 42 °C. Thereafter, cDNA was synthesized using a PrimeScriptTM RT reagent Kit (TaKaRa, Dalian, China) according to the manufacturer’s instructions and stored at − 20 °C until analysis.

Relative mRNA expression of FSHR, LSHR, ERα, ERβ, mPRα, mPRβ, 3β-HSD, 17β-HSD, CYP11A1, CYP19A1, CYP17A1, and StAR was conducted using a LightCycler480 system (Roche, Basel, Switzerland) with the SYBR® Premix EX TaqTM (TaKaRa, Dalian, China). The target and internal reference (GAPDH) genes primer sequences are detailed in Table 1 and were obtained from previously published papers^37^. Quantitative polymerase chain reaction (qPCR) parameters were as follows: denaturation 30 s at 95 °C, followed by 40 cycles of denaturation 5 s at 95 °C, and annealing 20 s at 60 °C. The specificity of the PCR reaction was confirmed by melting curve analysis. The efficiency of PCR amplification for each gene was checked against the dilution of the sample. The relative expression ratios of targeted genes were calculated by R = 2^−ΔΔCt method^38^, with the CT group used as the calibrator.

**Table 1:** The primer sequences of the target genes^36^.

Target genes^a^	Primer sequence (5' → 3')	Product length, bp	Accession No.^b^
GAPDH	Forward (F): ACCCAGAAGACTGTGGATGG	178	NM_001034034
	Reverse (R): CAACAGACACGTTGGGAGTG		
FSHR	F: GCCAGCCTCACCTACCCCAGC	75	NM_174061
	R: AATTGGATGAAGGTCAGAGGTTTGCC		
LHR	F: ATTGCCTCAGTCGATGCCCAGACC	92	NM_174381
	R: AAAAAGCCAGCCGCGCTGC		
ERα	F: TCAGGCTACCATTACGGAGTTT	120	NM_001001443
	R: TTCTGATCCTGCTGTTGAGAAA		
ERβ	F: CTTCGTGGAGCTCAGCCTGT	241	NM_174051
	R: GTTTTTATCAATCGTGCACTGG		
mPRα	F: CCGGCGGTCCATCTATGA	159	NM_001038553
	R: CCACCCCCTTCACTGAGTCTT		
mPRβ	F: TGCCCCTGCTCGTCTATGTC	120	NM_001101135
	R: CCCACGTAGTCCACGAAGTAGAA		
StAR	F: TTTTTTCCTGGGTCCTGACAGCGTC	103	NM_174189
	R: ACAACCTGATCCTTGGGTTCTGCACC		
3β-HSD	F:GCCACCTAGTGACTCTTTCCAACAGCG	111	NM_174343
	R: TGGTTTTCTGCTTGGCTTCCTCCC		
17β-HSD	F: CGCATATTGGTGACCGGGAGCATA	108	NM_001102365
	R: AATCGCCAGACTCTCGCACAAACC		
CYP11A1	F: CAGTGTCCCTCTGCTCAACGTCC	99	NM_176644
	R: TTATTGAAAATTGTGTCCCATGCGG		
CYP19A1	F: CGCCACTGAGTTGATTTTTGCTGAGA	301	NM_174305
	R: TAAGGCTTTGCGCATGACCAGGTC		
CYP17A1	F: GACAAAGGCACAGACGTTGTGGTCA	301	NM_174304
	R: TGATCTGCAAGACGAGACTGGCATG		

### Western blot analysis

To detect the key protein (17β-HSD, FSHR, LHR, StAR, ERα, ERβ, CYP17A1, *p*-AKT, AKT, *p*-mTOR, mTOR, *p*-PI3K, and PI3K) involved in gonadal hormones regulation, western blot was performed. Firstly, total protein was extracted using RIPA solution (Cell Signaling Technology, Inc., Danvers, MA), with the protease inhibitor cocktail (Roche, Penz-berg, Germany) and phosphatase inhibitor cocktail (Roche, Penz-berg, Germany) as described previously^11^. The protein concentration was determined using an enhanced BCA assay kit (Beyotime Biotechnology, Shanghai, China). Secondly, 30 μg protein samples were mixed with 5× loading buffer, denatured (100 °C, 5 min), and separated by 10% sodium dodecyl sulfate–polyacrylamide gel electrophoresis (SDS-PAGE) at 100 V for 30 min, followed by 120 V for about 1.5 h. The isolated protein was transferred into Polyvinylidene Fluoride membranes (Immobilon-FL membrane, Millipore, Danvers, MA) with 0.45 μm apertures at a constant 200 mA for 1.5 h at 4 °C. Nonspecific binding sites of membranes were blocked in 5% bovine serum albumin (BSA) and 0.1% Tris-Buffered-Saline with Tween (TBST) at room temperature for 2 h. Then, membranes were washed and incubated at 4 °C overnight with the specific primary antibodies (Table 2). After several washes in TBST, membranes were incubated with corresponding secondary antibodies (Table 2) for 2 h at room temperature. Finally, the blot was washed 3 times for 10 min with wash buffer, and bands were detected by enhanced chemiluminescence (ECL) using Luminata Classico Western HRP Substrate (WBLUC0100, Millipore, Danvers, MA).The ECL signals were recorded using an imaging system (Bio-Rad, California, USA) and quantified using Quantity One software (Bio-Rad, USA) normalized to GAPDH. In the bar graph, each column represents an average of four or five cows per group. A representative set of blots from six cows was shown here.

**Table 2:** Antibodies, suppliers and dilutions used for western blot analysis.

Items	Type	Suppliers	Dilution
Primary antibodies			
*p*-AKT	Rabbit polyclonal 4060	Cell Signaling Technology, Danvers, MA, USA	1/2000
AKT	Rabbit polyclonal 9272	Cell Signaling Technology, Danvers, MA, USA	1/1000
*p*-mTOR	Rabbit polyclonal 2971	Cell Signaling Technology, Danvers, MA, USA	1/1000
mTOR	Rabbit polyclonal ab2732	Abcam, Cambridge, UK	1/1500
*p*-PI3K	Rabbit polyclonal bs-5582R	Biosynthesis Biotechnology, Beijing, China	1/200
PI3K	Rabbit polyclonal	Biorbyt, Cambridge, UK	1/300
ERα	Rabbit polyclonal ab3575	Abcam, Cambridge, UK	1/500
ERβ	Rabbit polyclonal ab3577	Abcam, Cambridge, UK	1/500
FSHR	Rabbit polyclonal bs-0895R	Biosynthesis Biotechnology, Beijing, China	1/500
LHR	Rabbit polyclonal ab179780	Abcam, Cambridge, UK	1/500
StAR	Rabbit polyclonal Ab96637	Abcam, Cambridge, UK	1/1000
CYP17A1	Rabbit polyclonal orb5948	Biorbyt, Cambridge, UK	1/300
17β-HSD	Rabbit polyclonal bs-6603R	Biosynthesis Biotechnology, Beijing, China	1/500
GAPDH	Mouse monoclonal 60004-1-lg	Proteintech, USA	1/5000
Secondary antibodies			
Goat anti-rabbit IgG	SA00001-2	Proteintech, USA	1/3000
Donkey anti-mouse	SA00001-8	Proteintech, USA	1/3000

### Histological examination

Ovaries (n = 3 per group) were fixed in 10% buffered formalin, processed routinely through ascending alcohols and xylene, embedded in paraffin, serially sectioned at 5 µm, and stained with hematoxylin and eosin (HE)^39^. Follicle classification as primordial or growing was performed on HE-stained sections under an Eclipse TE2000U inverted microscope with a twin CCD camera (Nikon, Tokyo, Japan). Three sections of each tissue for each cow were detected^40^. In brief, to avoid counting a follicle twice, only follicles with a visible oocyte nucleus were counted. A primordial follicle was defined as an oocyte surrounded by a single layer of flattened pre-granulosa cells. Growth follicles include follicles at all stages of development. No atretic follicles were examined in this study. Two experimenters counted the number of follicles in each category without knowing the groups. Finally, the percentage of the number of follicles (primordial or growing) on the total number of oocytes was calculated.

### Immunohistochemical analysis

Sections of each ovary sample (slides: three for every group) were made for Immunohistochemical (IHC) examinations of FSHR and LHR using a previously described method^41^. Briefly, the sections were covered with 0.5% TritonX-100 in phosphate buffer saline (PBS) for 20 min. Endogenous peroxidase signals were blocked with 3% hydrogen peroxide in methanol for 5 min at room temperature. 5% bovine serum albumin (BSA: Sigma-Aldrich, Cat: A9647) diluted in 0.1 M PBS (pH 7.2) was utilized to block the non-specific background staining for 1 h, and incubated with anti-FSHR and anti-LHR antibodies overnight at 4 °C. The primary antibody for each protein was diluted in PBS (PH = 7.4) at the following dilutions: FSHR (Biosynthesis Biotechnology, Beijing, China) 1:400; LHR (Biosynthesis Biotechnology, Beijing, China) 1:400. In negative control sections, PBS substituted the primary antibody-containing solution. On the following day, the sections were cleaned 3 times in PBS for 5 min, incubated with biotinylated anti-rabbit IgG antibody (for FSHR and LHR, Birmingham, USA) diluted 1:200 in PAV buffer (containing 0.1 M PBS, 0.1% BSA, and 0.05% thimerosal) for 50 min at 4 °C. Thereafter, the sections were washed 3 times in PBS for 5 min, and visualized using diaminobenzidine tetrahydrochloride (DAB). Next, they were rinsed and counterstained with Mayer's hematoxylin. Then, the sections were dehydrated and covered with a mounting medium (DPX; POCh, Gliwice, Poland). Finally, the sections were examined by two independent observers using an optical microscope. Semi-quant was assessed to estimate the expression level of LHR and FSHR in the ovary for every group taking advantage of the software Image-pro plus 6.0 (Media Cybernetics, Washington, USA)^42^. Approximately 3 fields were examined per slide, and 3 slides were analyzed per group. Ovarian tissue exposed to secondary antibodies only was used as a negative control.

### Statistical analysis

All data were analyzed by using SPSS 21.0 (Chicago, IL, USA). For postpartum plasma hormone concentrations, the data were analyzed by a two-way analysis of variance, where the fixed effects were the RPG treatment, collection time, and RPG treatment by collection time interactions, the individual was considered as a random effect. The collection time was considered a repeated measure. Meanwhile, the Mann–Whitney U test was used to assess the difference between the CT and RPG groups at each time point. The data were presented as Box and Whisker Plots, with an overlap of scatter plots. For the data on ovarian weight, ovarian index, and ovarian gene and protein expressions, the Mann–Whitney U test was used to assess the difference between the CT and RPG groups. And the data were presented as Box and Whisker Plots, with an overlap of scatter plots. For the data of histology and immunohistochemistry of the ovary, the Mann–Whitney U test was used to assess the difference between the means. G power analysis of hormones was performed using *Gpower* software (version 3.0.10). And the data were presented as the mean ± standard error of the mean (SEM). Statistical significance was considered significant when *P* < 0.05, and 0.05 < *P* < 0.1 was considered a tendency.

## Supplementary Information

Supplementary Information.

## Data Availability

All data associated with this study are present in the paper or the Supplementary Materials.
