# Supplementary material for: Rumen-protected glucose stimulates the secretion of reproductive hormones and the mTOR/AKT signaling pathway in the ovaries of early postpartum
Source: Sci Rep. 2023 Feb 20;13:2940. doi: 10.1038/s41598-023-30170-5 (PMC9941576; doi:10.1038/s41598-023-30170-5)
Supplement: Supplementary file 1 — Supplementary Information. [file 41598_2023_30170_MOESM1_ESM.docx]

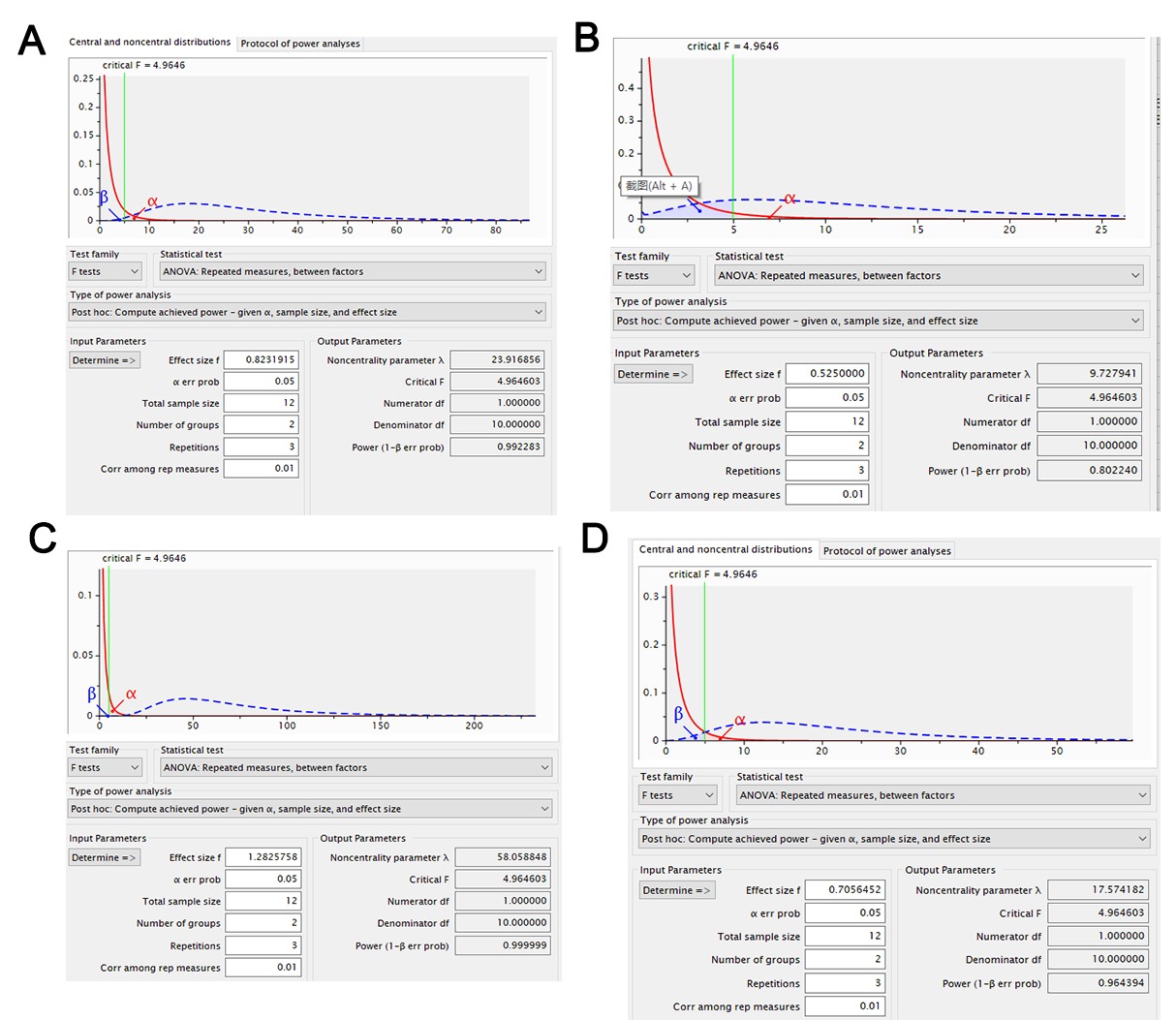


**Supplementary Figure 1.** The statistical power of E2 (A), P4 (B), FSH (C), and LH (D) in 12 cows were calculated by GPower software.


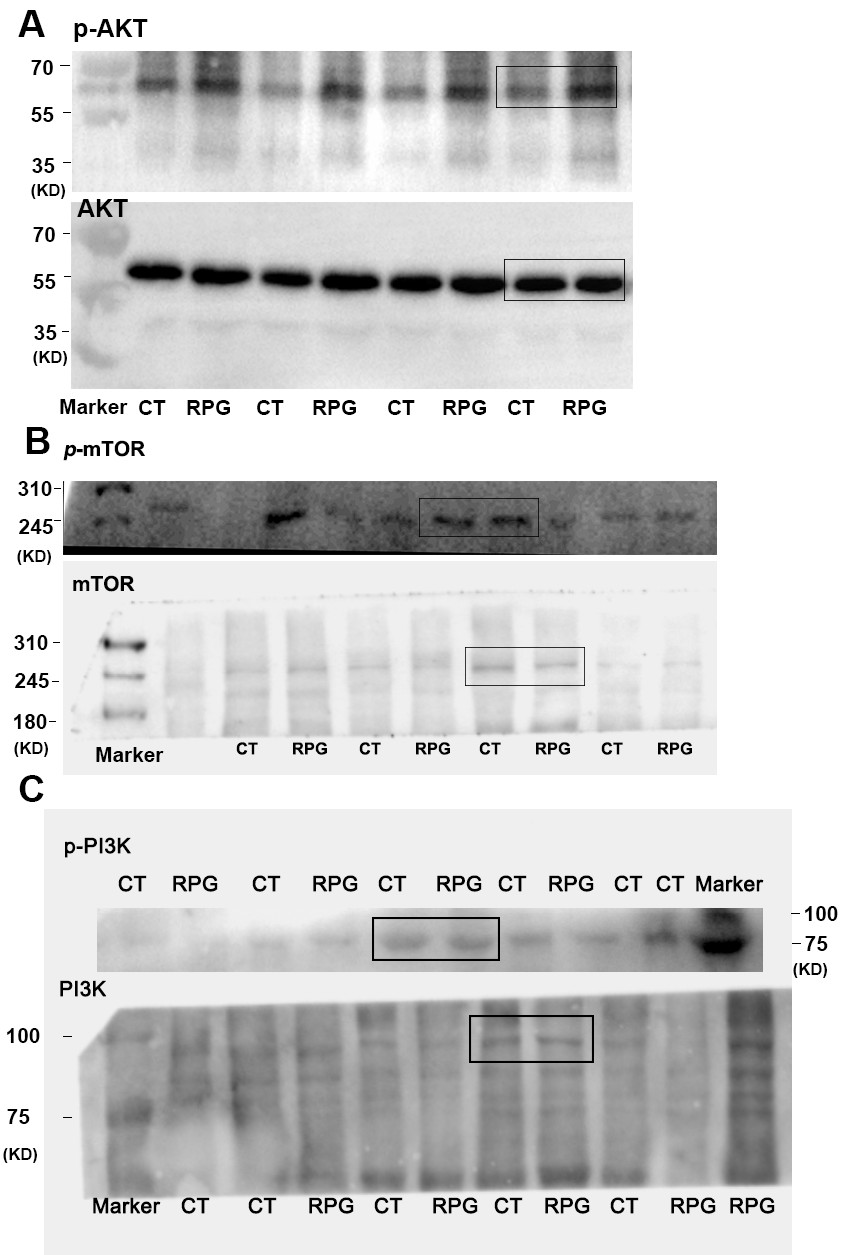


**Supplementary Figure 2.** The black box shows the graphical section in Figure 4 from manuscript

**
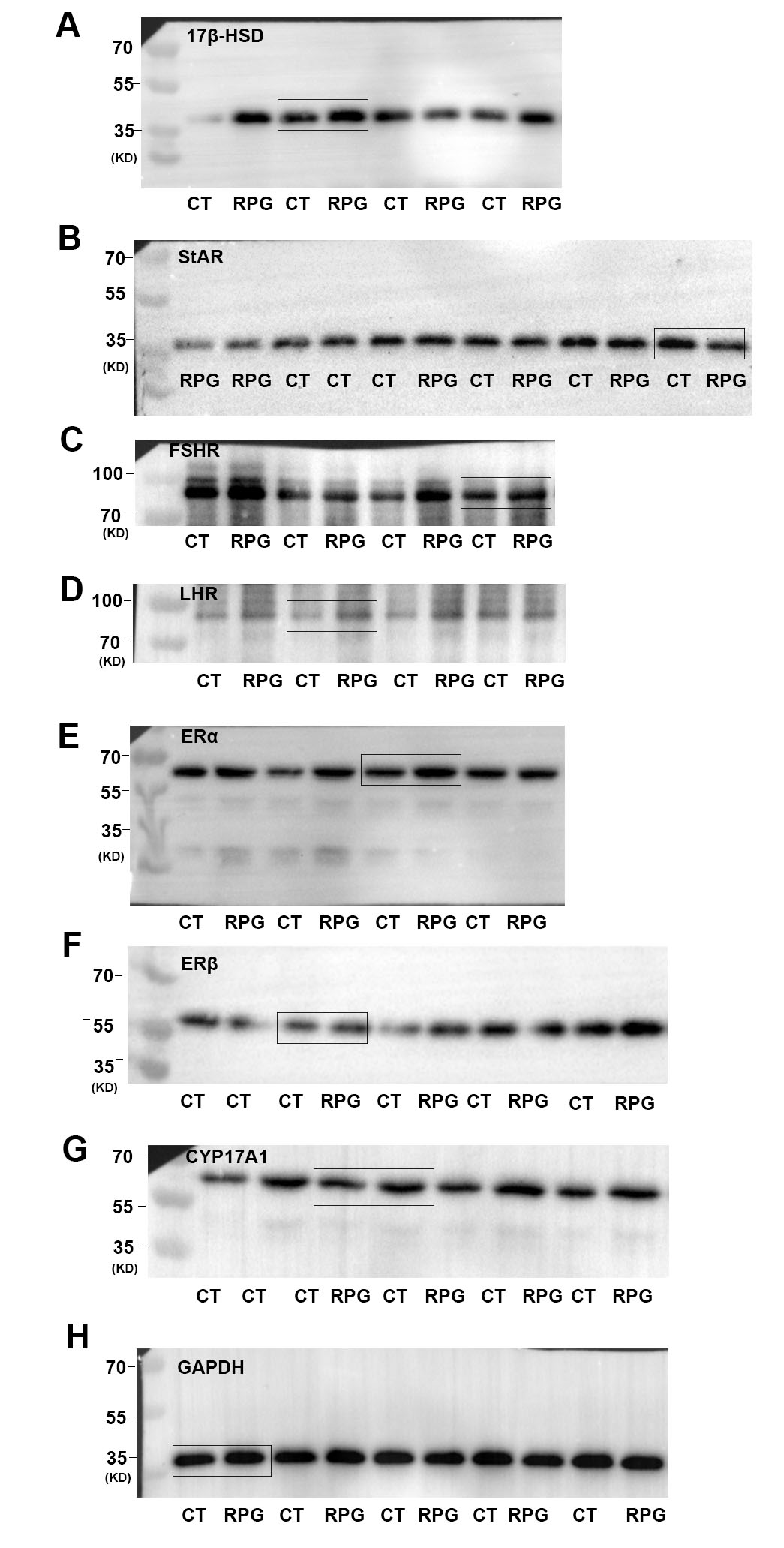
**

**Supplementary Figure 3.** The black box shows the graphical section in Figure 5 from manuscript.

**Supplementary Table 1. Ingredients and nutrient composition of the diets.**

| **Item** | **Prepartum** | **Postpartum** |
| --- | --- | --- |
| Ingredient (% of DM) |  |  |
| Corn silage | 24.7 | 30.2 |
| Oat hay | 55.3 | 12.8 |
| Alfalfa hay | - | 17.1 |
| Ground shelled corn | 5.9 | 7.0 |
| Wheat bran | 9.1 | 21.3 |
| Soybean meal (49% CP) | 4.1 | 8.1 |
| Calcium carbonate | 0.23 | 1.13 |
| Calcium hydrophosphate | 0.23 | 0.45 |
| Salt | 0.16 | 0.45 |
| Sodium bicarbonate | - | 0.68 |
| Magnesium oxide | 0.05 | 0.09 |
| Potassium chloride | - | 0.32 |
| Vitamin and mineral mix ^1^ | 0.23 | 0.41 |
| Chemical analysis, % of DM |  |  |
| CP | 11.6 | 14.6 |
| Fat | 2.0 | 2.1 |
| Starch | 10.3 | 14.8 |
| NDF | 53.6 | 45.2 |
| ADF | 31.3 | 25.5 |
| Ash | 6.8 | 6.0 |
| Ca | 0.50 | 0.98 |
| P | 0.41 | 0.54 |
| NE_L_ ^2^, Mcal/kg | 1.30 | 1.37 |

^1^ Dry cow vitamin and mineral premix (per kg): Cu 900 mg; Zn 1350 mg; Mn 1000 mg; Co 13 mg; I 27 mg; Se 23 mg; vitamin A 450 KIU; vitamin D3 110 KIU; vitamin E 5400 IU. Lactating cow vitamin and mineral premix (per kg): Cu 3040 mg; Fe 3170 mg; Zn 14,280 mg; Mn 3060 mg; Co 40 mg; I 180 mg; Se 100 mg; vitamin A 1250 KIU; vitamin D 3270 KIU; vitamin E 5000 IU. ^2^ Calculated value.
